# Supplementary material for: Spironolactone is an antagonist of NRG1‐ERBB4 signaling and schizophrenia‐relevant endophenotypes in mice
Source: EMBO Mol Med. 2017 Jul 25;9(10):1448–62. doi: 10.15252/emmm.201707691 (PMC5653977; doi:10.15252/emmm.201707691)
Supplement: Supplementary file 1 — Appendix [file EMMM-9-1448-s001.pdf]

Appendix

Michael C. Wehr, Wilko Hinrichs, Magdalena M. Brzózka, Tilmann Unterbarnscheidt, Alexander Herholt, Jan P. Wintgens, Sergi Papiol, M. Clara Soto-Bernardini, Mykola Kravchenko, Mingyue Zhang, Klaus-Armin Nave, Sven P. Wichert, Peter Falkai, Weiqi Zhang, Markus H. Schwab & Moritz J. Rossner

Spironolactone is an Antagonist of NRG1-ERBB4 Signaling and Schizophrenia-Relevant Endophenotypes in Mice

Table of content

Appendix Fig S1. Summary of the validation strategy for drug repurposing performed on the NRG1-ERBB4-PI3K schizophrenia risk pathway.

Appendix Fig S2. Protein expression analysis reveals strong Erbb4, but no detectable Egfr expression in the adult mouse brain.

Appendix Fig S3. Covariate analysis revealed no significant effect of age on the treatment response.

Appendix Table S1. Final hit list of approved drugs.

Appendix Table S2. Oligonucleotides used for cloning.

Appendix Table S3. List of antibodies used.

Appendix Supplementary Reference.

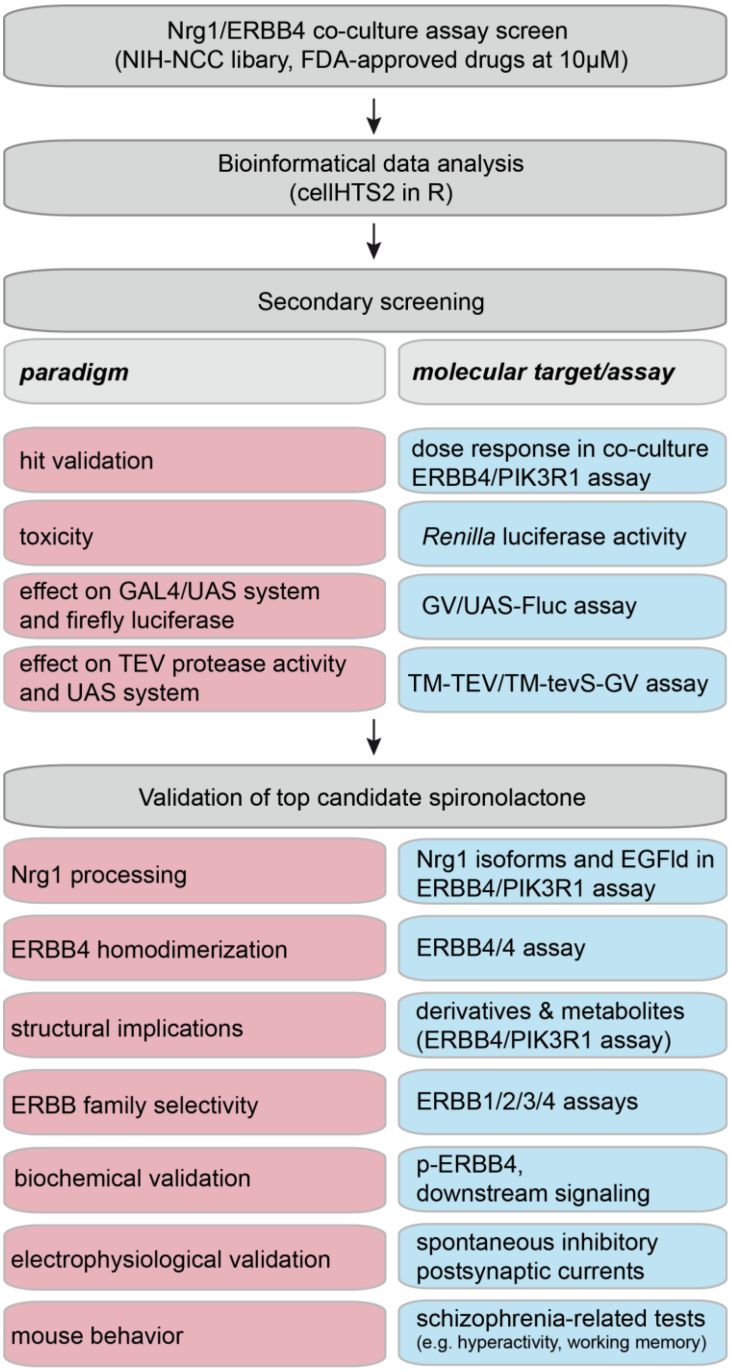

Appendix Fig S1. Summary of the validation strategy for drug repurposing performed on the NRG1-ERBB4-PI3K schizophrenia risk pathway.

To identify small molecule modulators of NRG1-ERBB4 signaling in the context of drug repurposing and schizophrenia, the NIH-NCC compound collection was subjected to a cell-based co-culture assay screening approach using the split TEV technique as readout. Hits recovered from the screen were analyzed using the computational method cellHTS2 and validated using a consecutive analysis of various individual assays as depicted. The paradigm tested is shown on the left, the molecular target assessed or assay applied on the right.

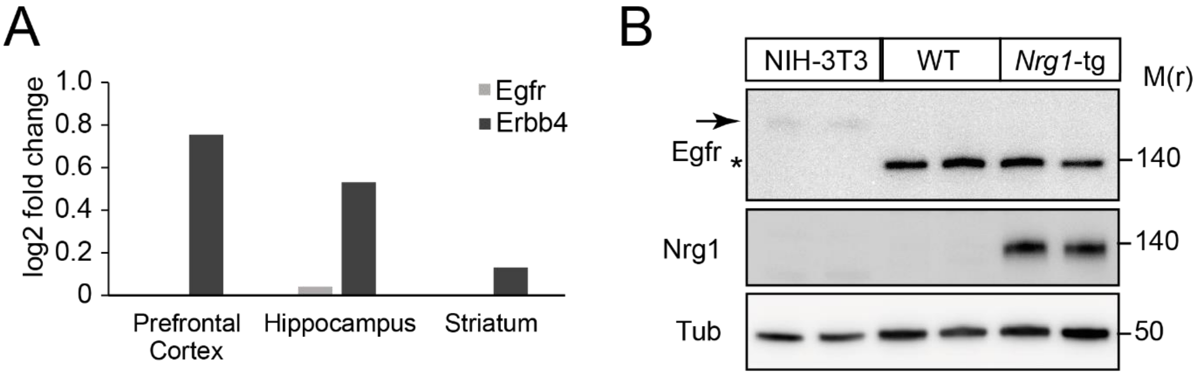

Appendix Fig S2. Protein expression analysis reveals strong Erbb4, but no detectable Egfr expression in the adult mouse brain.

(**A**) Erbb4 protein expression is increased in prefrontal cortex, hippocampus and striatum. Expression data is normalized to overall protein expression and values are log2-transformed, positive values are plotted. Data is extracted from http://www.mousebrainproteome.com/ (Sharma *et al*, 2015).

(**B**) Egfr is not expressed in prefrontal cortex of adult WT and *Nrg1*-tg mice. Lysates from NIH-3T3 cells (fibroblast-derived mouse cell line with low Egfr expression) and prefrontal cortex (both WT and *Nrg1*-tg mice, P60) were subjected to Western Blotting and probed for indicated antibodies. Arrow indicates band representing Egfr; asterisk indicates an unspecific band.

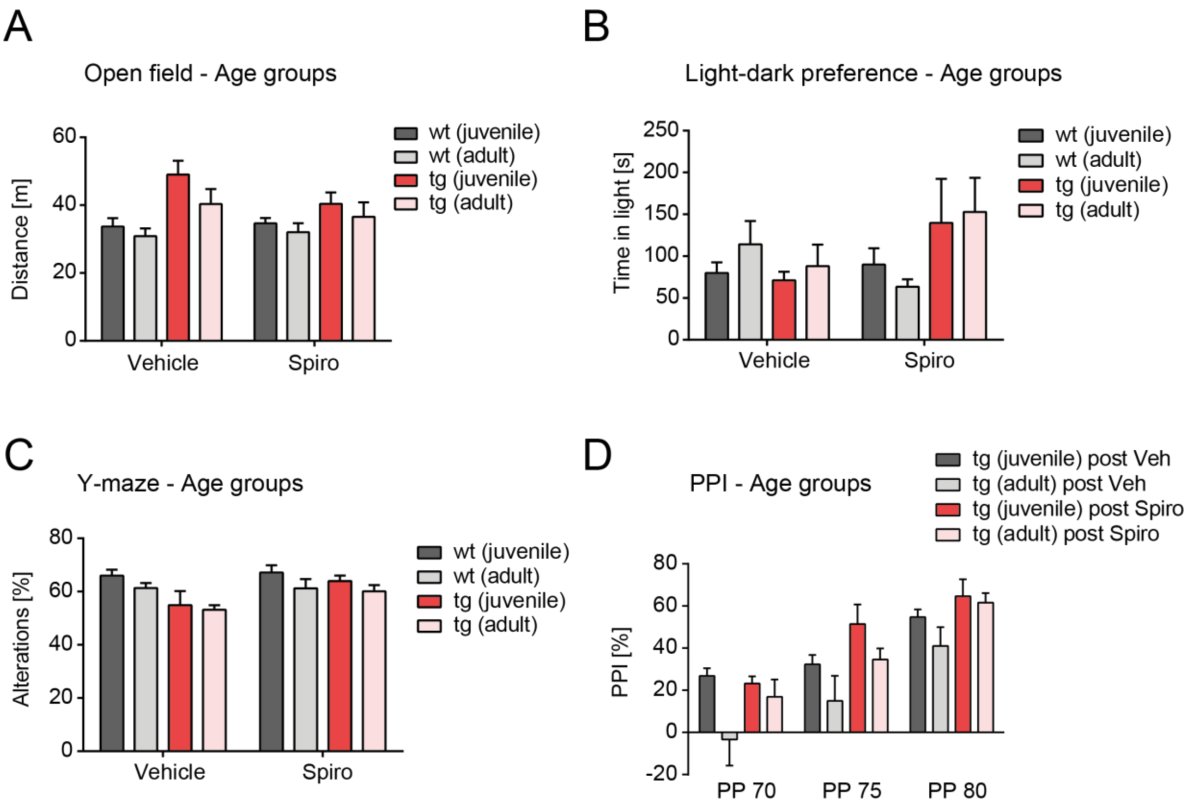

Appendix Fig S3. Covariate analysis revealed no significant effect of age on the treatment response.

(**A**) Two-way ANCOVA considering age groups (juvenile/adult) as covariate did not report a significant genotype x treatment interaction ($F_{(1,43)}=2.174$; $p=0.148$). Related to Fig 5B. There were no overall differences between juvenile and adult mice within each experimental condition (all t-tests $p\geq0.199$).

(**B**) Two-way ANCOVA considering age groups (juvenile/adult) as covariate reported a significant genotype x treatment interaction ($F_{(1,40)}=4.838$; $p=0.034$). Related to Fig 5E. Absence of statistically significant age-related effects on the light-dark preference test. There were no overall differences between juvenile and adult mice within each experimental condition (all t-tests $p\geq0.221$).

(**C**) Two-way ANCOVA considering age groups (juvenile/adult) as covariate reported a suggestive genotype x treatment interaction ($F_{(1,43)}=3.528$; $p=0.067$). Related to Fig 5F. There were no overall differences between juvenile and adult mice within each experimental condition (all t-tests $p\geq0.209$).

(**D**) Pairwise analyses revealed also for PPI no significant differences between juvenile and adult mice within each experimental condition (all t-tests $p\geq0.107$). Related to Fig 5G.

Data are shown as mean, error bars represent SEM. *, $p<0.05$; Spiro, spironolactone; Veh, vehicle; juvenile mice were defined as 8-11 weeks old, adult mice were defined as 12-16 weeks old.

For (A) and (C), juvenile *Nrg1*-tg vehicle, n=5, adult *Nrg1*-tg vehicle, n=7; juvenile *Nrg1*-tg Spiro, n=5; adult *Nrg1*-tg Spiro, n=7; juvenile wt Vehicle, n=8; adult wt vehicle, n=4; juvenile wt Spiro, n=7; adult wt Spiro, n=5.

For (B), juvenile *Nrg1*-tg vehicle, n=5, adult *Nrg1*-tg vehicle, n=6; juvenile *Nrg1*-tg Spiro, n=4; adult *Nrg1*-tg Spiro, n=6; juvenile wt Vehicle, n=8; adult wt vehicle, n=4; juvenile wt Spiro, n=7; adult wt Spiro, n=5;

For (D), juvenile *Nrg1*-tg vehicle, n=4, adult *Nrg1*-tg vehicle, n=7; juvenile *Nrg1*-tg Spiro, n=5; adult *Nrg1*-tg Spiro, n=7.

Appendix Table S1. Final hit list of approved drugs.

Rank	Z-score	NCC_Sample_ID	Pubchem_CID	Compound_name, synonyms
1	9.25	SAM002264648	5833	Spironolactone
2	7.97	SAM001246651	60699	Topotecan HCl
3	6.59	SAM001246973	2690	CGS 15943
4	6.52	SAM002264631	4828	Pindolol
5	5.88	SAM001246964	23581797	3'-Deoxydenosine
6	5.88	SAM001246573	23581791	Vardenafil citrate
7	4.7	SAM001246669	2161	Amlexanox
8	4.3	SAM002699899	5362129	Ramipril
9	4.26	SAM001246548	3749	Irbesartan
10	3.82	SAM002589959	3003	Betamethasone
11	3.13	SAM001246805	11693521	Telithromycin
12	3	SAM002548951	1349907	Methimazole
13	-3.6	SAM001246679	3793	Itraconazole
14	-3.5	SAM001246526	20279	2-Chloro-2'-deoxyadenosine
15	-3.45	SAM002264605	65327	Norpramin
16	-3.45	SAM001246775	2378	Bifonazole
17	-4.63	SAM002548959	4030	Mebendazole
18	-5.48	SAM002589939	2082	Albendazole

The final hit list contains candidates that score at least 3 standard deviations (measured as z-score) from the mean. Screening data for all compounds can be found the accompanying file Dataset EV1.

Appendix Table S2. Oligonucleotides used for cloning.

Oligonucleotide name	Sequence
GRB2_B1-Kozak	GGGGA<u>CAAGTTTGTACAAAAAAGCAG</u>GCTCCACCATGGAAGCCATCGCCAAATATGACTTC
GRB2_B2	GGGGA<u>CCACTTTGTACAAGAAAGCTG</u>GGTCGACGTTCCGGTTCACGGGG
PIK3R1_B1-Kozak	GGGGA<u>CAAGTTTGTACAAAAAAGCAG</u>GCTCCACCATGAGTGCTGAGGGGTACCAGTAC
PIK3R1_B2	GGGGA<u>CCACTTTGTACAAGAAAGCTG</u>GGTCTCGCCTCTGCTGTGCATATACTGG
SHC1_B1-Kozak	GGGGA<u>CAAGTTTGTACAAAAAAGCAG</u>GCTCCACCATGGATCTCCTGCCCCCCAAG
SHC1_B2	GGGGA<u>CCACTTTGTACAAGAAAGCTG</u>GGTCCAGTTTCCGCTCCACAGGTTGC
SRC_B1-Kozak	GGGGA<u>CAAGTTTGTACAAAAAAGCAG</u>GCTCCACCATGGGTAGCAACAAGAGCAAGCCC
SRC_B2	GGGGA<u>CCACTTTGTACAAGAAAGCTG</u>GGTCGAGGTTCTCCCCGGGCTGG
STAT5A_B1-Kozak	GGGGA<u>CAAGTTTGTACAAAAAAGCAG</u>GCTCCACCATGGCGGGCTGGATCCAGG
STAT5A_B2	GGGGA<u>CCACTTTGTACAAGAAAGCTG</u>GGTCTGAGAGGGAGCCTCTGGCAG
ERBB4_B1-Kozak	GGGGA<u>CAAGTTTGTACAAAAAAGCAG</u>GCTCTACCATGAAGCCGGCGACAGGACTTTGG
ERBB4_B2	GGGGA<u>CCACTTTGTACAAGAAAGCTG</u>GGTCCACCACAGTATTCCGGTGTCTGTAAG
ERBB4_1_685_B2	GGGGA<u>CCACTTTGTACAAGAAAGCTG</u>GGTCGGCTCTTTTCTTTTTGATGC

The oligonucleotides listed above were used for cloning of open reading frames (ORFs) using Gateway recombination cloning (attB1 and attB2 sites are underlined). The oligonucleotide names contain the HGNC nomenclature of human ORFs cloned.

Appendix Table S3. List of antibodies used.

Antibody	Species	Dilution	Experiment	Source	Catalog Number
Akt	rabbit	1:1000	WB	Cell Signaling Technology	9272
EGFR (A-10)	mouse	1:500	WB	Santa Cruz Biotechnology	sc-373746
ErbB4 (E200)	rabbit	1:1000	WB	Abcam	ab32375
HA (clone 3F10)	rat	1:500	ICC	Roche	11 867 423 001
LIMK1	rabbit	1:1000	WB	Cell Signaling Technology	3842
Neuregulin-1α/β1/2 (C-20)	rabbit	1:1000	WB	Santa Cruz Biotechnology	sc-348
p44/42 MAPK (Erk1/2) (137F5)	rabbit	1:2000	WB	Cell Signaling Technology	4695
Phospho-Akt (Ser473) (D9E)	rabbit	1:1000	WB	Cell Signaling Technology	4060
p-ErbB-4 (Tyr 1056)	rabbit	1:500	WB	Santa Cruz Biotechnology	sc-33040
Phospho-HER4/ErbB4 (Tyr1284) (21A9)	rabbit	1:500	WB	Cell Signaling Technology	4757S
Phospho-LIMK1 (Thr508)/LIMK2 (Thr505)	rabbit	1:500	WB	Cell Signaling Technology	3841
Phospho-p44/42 MAPK (Erk1/2) (Thr202/Tyr204) (D13.14.4E)	rabbit	1:2000	WB	Cell Signaling Technology	4370
Tubulin	mouse	1:2000	WB	Sigma	T 5168

WB, Western Blot; ICC, immunocytochemistry.

Appendix Supplementary Reference

Sharma K, Schmitt S, Bergner CG, Tyanova S, Kannaiyan N, Manrique-Hoyos N, Kongi K, Cantuti L, Hanisch U-K, Philips M-A, Rossner MJ, Mann M & Simons M (2015) Cell type- and brain region-resolved mouse brain proteome. *Nat. Neurosci.* **18** 1819–1831
