# Supplementary material for: Spironolactone is an antagonist of NRG1‐ERBB4 signaling and schizophrenia‐relevant endophenotypes in mice
Source: EMBO Mol Med. 2017 Jul 25;9(10):1448–62. doi: 10.15252/emmm.201707691 (PMC5653977; doi:10.15252/emmm.201707691)
Supplement: Supplementary file 2 — Expanded View Figures PDF [file EMMM-9-1448-s002.pdf]

Expanded View Figures

Figure EV1. A co-culture assay based on the split TEV technique to monitor NRG1-ERBB4 signaling activity.

A PC12 cell lines stably expressing mouse Nrg1 type I β1a and Nrg1 type III β1a. PC12 cells were stably transfected with a plasmid encoding Nrg1 type I β1a or type III β1a, and Nrg1 expression was verified by Western blot analysis using an Nrg1 antibody that detects the β1a C-terminus.

B Co-culturing two populations of PC12 cells. Cell population A was transfected with a nuclear localized EYFP (EYFPnuc), and cell population B was transfected with ECFP. Following to an initial expression of 24 h, cells were mixed and imaged 24 h later. Scale bar, 100 μm.

C ERBB4-PIK3R1 dose–response assay using EGFld as stimulus. Three independent experiments using the ERBB4-PIK3R1/EGFld assay are shown. The EC_{50} value for this assay is at 1 ng/ml EGFld. The inset depicts the Z' factors for each individual assay.

D Lapatinib antagonizes ERBB4-PIK3R1 signaling in a dose-dependent manner. Per 96-well, 40,000 split TEV assay cells were incubated with increasing amounts of lapatinib, followed by a stimulation using 10 ng/ml EGFld.

E, F CI-1033 antagonizes ERBB4-PIK3R1 signaling in a dose-dependent manner. Per 96-well, 40,000 split TEV assay cells were incubated with increasing amounts of CI-1033, followed by either co-plating 10,000 Nrg1-expressing cells (E) or stimulation with 10 ng/ml EGFld (F).

Data information: Fluc, firefly luciferase activity (black lines); Rluc, *Renilla* luciferase activity (gray lines, indicating toxicity levels). Data are shown as mean, and error bars represent SEM, $n = 6$. The insets depict IC_{50} values in μM.
Source data are available online for this figure.

 © 2017 The Authors

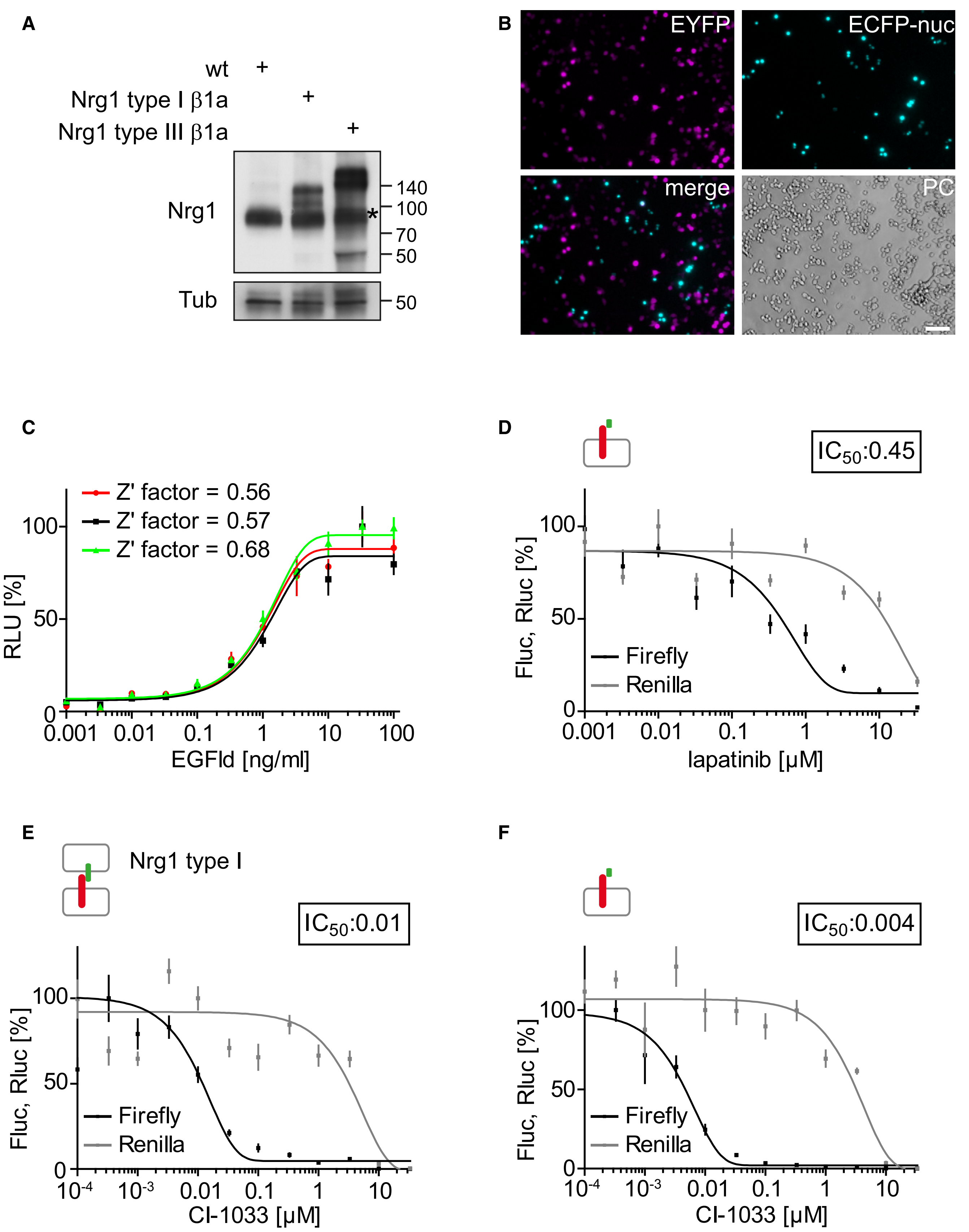
A
wt
Nrg1 type I β1a
Nrg1 type III β1a
Nrg1
Tub
140
100
70
50
50
B
EYFP
ECFP-nuc
merge
PC
C
Z' factor = 0.56
Z' factor = 0.57
Z' factor = 0.68
RLU [%]
EGFld [ng/ml]
D
IC50:0.45
Fluc, Rluc [%]
Firefly
Renilla
lapatinib [μM]
E
Nrg1 type I
IC50:0.01
Fluc, Rluc [%]
Firefly
Renilla
CI-1033 [μM]
F
IC50:0.004
Fluc, Rluc [%]
Firefly
Renilla
CI-1033 [μM]

Figure EV1.

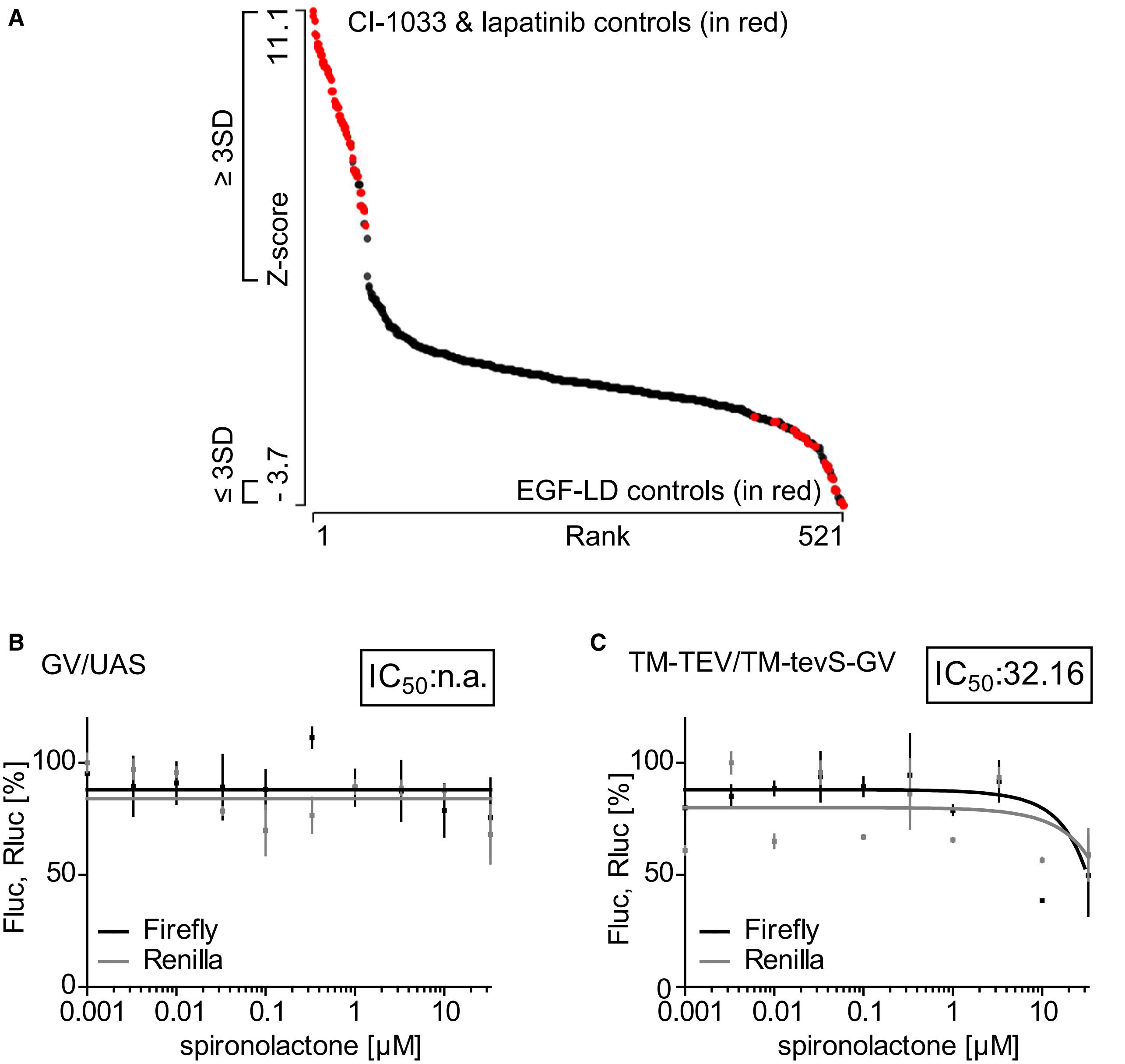

Figure EV2. Spironolactone is the top candidate recovered from the co-culture screen.

A Graphic visualization of the primary screen data of the NIH-NCC library, set 2. All counts (407 compounds and 96 controls) from the Nrg1-ERBB4-PIK3R1 split TEV compound screen were plotted against the *z*-score using Mondrian, with pathway activators displaying high values and inhibitors low values. For the secondary analysis, we selected all candidates that were at least three standard deviations away from the mean. EGFld-positive and lapatinib/CI-1033-negative controls are shown in red.

B, C Spironolactone does not affect activities of control assays. Neither the activity of the GAL4/UAS system (B) nor the activity of the TEV protease (C) was affected in spironolactone dose-dependent assays. For the GAL4/UAS assay, GAL4-VP16 (GV) and 10×UAS firefly luciferase (10×UAS-Fluc) plasmids were transfected into PC12 cells. For TEV protease activity assays, a transmembrane-localized TEV protease (TM-TEV) was expressed in PC12 cells with a transmembrane-localized and TEV-cleavable GV (TM-tevS-GV) and the 10×UAS-Fluc reporter. 10 ng/µl EGFld was applied as Nrg1 stimulus. In both control assays, the assay activity (firefly luciferase, black lines) was similar to the toxicity levels (gray lines). Data are shown as mean, a nd error bars represent SEM, n = 6. The insets depict IC_{50} values in µM.

 © 2017 The Authors

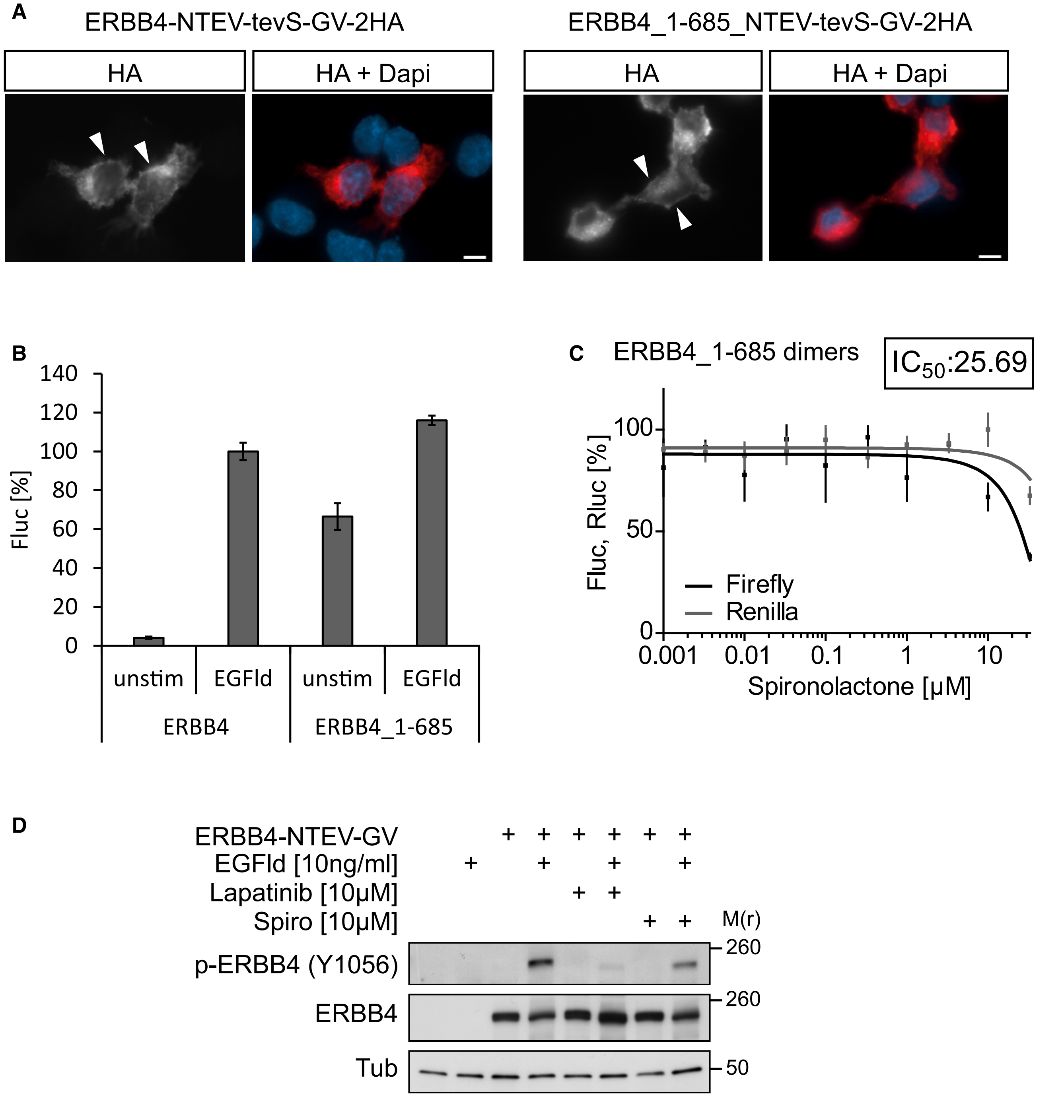
A
ERBB4-NTEV-tevS-GV-2HA
ERBB4_1-685_NTEV-tevS-GV-2HA
HA
HA + Dapi
B
Fluc [%]
140
120
100
80
60
40
20
0
unstim
EGFld
ERBB4
ERBB4_1-685
C
ERBB4_1-685 dimers
IC50:25.69
Fluc, Rluc [%]
Firefly
Renilla
0.001
0.01
0.1
1
10
Spironolactone [µM]
D
ERBB4-NTEV-GV
EGFld [10ng/ml]
Lapatinib [10µM]
Spiro [10µM]
M(r)
p-ERBB4 (Y1056)
ERBB4
Tub
260
50

Figure EV3.

Figure EV3. Truncated ERBB4 dimers are not inhibited by spironolactone.

A Full-length and truncated ERBB4 split TEV fusions localize to the cell membrane. PC12 cells were transfected with a full-length human ERBB4 split TEV fusion (ERBB4-NTEV-tevS-GV-2HA) (left panels) or a truncated human ERBB4 split TEV fusion (ERBB4_1–685_NTEV-tevS-GV-2HA) that covers residues 1–685 and contains the transmembrane domain but lacks the intracellular domain (right panels). Cells were allowed to express the fusion proteins for 24 h, fixed, and stained for the double HA (2HA) tag. Arrowheads indicate expression of full-length and truncated ERBB4 at the membrane. Scale bars, 5 μm.

B Both full-length and truncated ERBB4 dimerize in split TEV assays when stimulated with extracellular EGFld. PC12 cells were transfected with split TEV pairs of ERBB4-NTEV-tevS-GV/-CTEV and ERBB4_1–685_NTEV-tevS-GV/-CTEV, starved overnight, stimulated with EGFld (10 ng/ml) or mock-stimulated (unstim), incubated for 20 h, lysed, and subjected to a luciferase assay. Fluc, firefly luciferase activity.

C Spironolactone-mediated inhibition of the ERBB4 dimerization requires the intracellular domain. Truncated ERBB4 receptor molecules (amino acids 1–685) were fused to N- and CTEV moieties, transfected into PC12 cells and assayed in a spironolactone dose-dependent assay in the presence of 10 ng/ml EGFld using the split TEV technique. Spironolactone had no inhibitory effect, as the assay activity (Fluc, firefly luciferase, black line) was comparable to the toxicity levels (Rluc, *Renilla* luciferase, gray line). The inset depicts the IC_{50} value in μM.

D Spironolactone reduces ERBB4 levels. PC12 cells were transfected with the split TEV assay plasmid ERBB4-NTEV-tevS-GV (where indicated), stimulated with 10 ng/ml EGFld, 10 μM lapatinib, and 10 μM spironolactone for 1 h as indicated. Cell lysates were probed for ERBB4 phosphorylation levels at Tyr1056. Note that spironolactone reduces but not abolishes the phosphorylation levels.

Data information: Data are shown as mean, and error bars represent SEM, $n = 6$.
Source data are available online for this figure.

Figure EV4. Collection of ERBB dimer dose–response assays assessing the target specificity of spironolactone treatment.

A–J Fluc, firefly luciferase activity reporting ERBB dimer assay activity (black lines) using the split TEV assay technique; Rluc, *Renilla* luciferase activity (gray lines) assessing viability. EGFld (10 ng/ml) was applied as stimulus unless stated otherwise. Data are shown as mean, and error bars represent SEM, $n = 6$. The insets depict IC_{50} values in μM. The following combinations were tested
(X-NTEV-tevS-GV with Y-CTEV): (A) ERBB1 (EGFR)/ERBB1, (B) ERBB1/ERBB2, (C) ERBB1/ERBB3, (D) ERBB1/ERBB4, (E) ERBB2/ERBB2, (F) ERBB2/ERBB3, (G) ERBB2/ERBB4, (H) ERBB3/ERBB3, (I) ERBB3/ERBB4, (J) EGFR/EGFR with 100 ng/ml EGF as stimulus.

 © 2017 The Authors

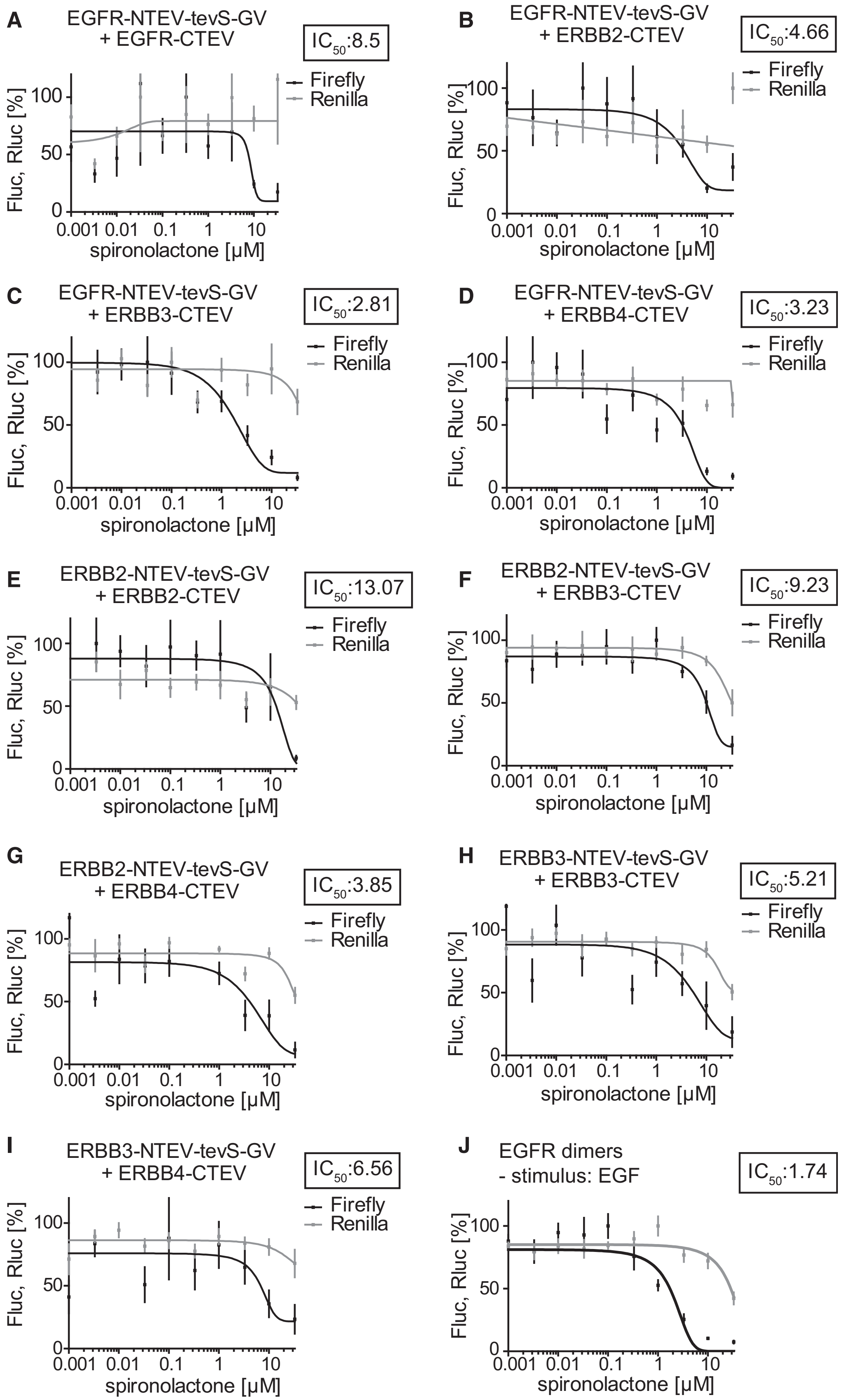
A
EGFR-NTEV-tevS-GV
+ EGFR-CTEV
IC50:8.5
B
EGFR-NTEV-tevS-GV
+ ERBB2-CTEV
IC50:4.66
C
EGFR-NTEV-tevS-GV
+ ERBB3-CTEV
IC50:2.81
D
EGFR-NTEV-tevS-GV
+ ERBB4-CTEV
IC50:3.23
E
ERBB2-NTEV-tevS-GV
+ ERBB2-CTEV
IC50:13.07
F
ERBB2-NTEV-tevS-GV
+ ERBB3-CTEV
IC50:9.23
G
ERBB2-NTEV-tevS-GV
+ ERBB4-CTEV
IC50:3.85
H
ERBB3-NTEV-tevS-GV
+ ERBB3-CTEV
IC50:5.21
I
ERBB3-NTEV-tevS-GV
+ ERBB4-CTEV
IC50:6.56
J
EGFR dimers
- stimulus: EGF
IC50:1.74
Firefly
Renilla
Fluc, Rluc [%]
0
50
100
0.001
0.01
0.1
1
10
spironolactone [µM]

Figure EV4.

Figure EV5. Behavioral analysis of *Nrg1*-tg and wt mice upon spironolactone treatment.

A Experimental design to test PPI deficits in *Nrg1*-tg mice. *Nrg1*-tg mice and wt controls were tested in the PPI test 1 day prior to chronic spironolactone treatment. One day after the last injection, transgenic and control mice were submitted to the PPI paradigm.

B Naïve *Nrg1*-tg displayed strong PPI deficits prior to chronic spironolactone treatment (effect of genotype $F_{1,33}$ = 25.74; $P < 0.0001$, two-way ANOVA, and Bonferroni *post hoc* test: ***$P < 0.0001$, ***$P = 0.0002$, ***$P = 0.0003$ for prepulse intensities 70, 75 and 80 dB, respectively).

C Chronic treatment with spironolactone improved PPI in transgenic animals (effect of treatment $F_{1,20}$ = 9.63; $P = 0.0056$, two-way ANOVA), with the most prominent effect when a prepulse of 70 dB was presented (**$P = 0.0060$, Bonferroni test; 75 and 80 dB were $P = 0.3067$ and $P = 0.1077$, respectively, Bonferroni test).

D Spironolactone treatment did not influenced PPI in wt controls (effect of treatment $F_{1,26}$ = 0.14; $P = 0.7096$, two-way ANOVA).

E *Nrg*-tg mice spent less time in the center of the open-field arena (effect of genotype $F_{1,44}$ = 6.69; $P = 0.0131$, two-way ANOVA). Spironolactone treatment did not influence this parameter ($F_{1,44}$ = 0.12; $P = 0.7290$, two-way ANOVA).

F In the open-field test, *Nrg1*-tg mice showed increased defecation (effect of genotype $F_{1,44}$ = 10.35; $P = 0.0024$, two-way ANOVA), which was significant in vehicle-treated animals (*$P = 0.0310$; Bonferroni test), but not in spironolactone-treated animals ($P = 0.0967$). There was no effect of treatment observed ($F_{1,44}$ = 0.65; $P = 0.4256$, two-way ANOVA).

G Transgenic animals displayed more frequent urination than wt controls (effect of genotype $F_{1,44}$ = 14.14; $P = 0.0005$, two-way ANOVA) in both vehicle and spironolactone-treated animals (*$P = 0.0451$ for vehicle-treated and *$P = 0.0100$ for spironolactone-treated groups, Bonferroni test), without effect of treatment ($F_{1,44}$ = 0.17; $P = 0.6781$, two-way ANOVA).

H In the light–dark preference test, transgenic mice performed similar number of transitions between light and dark compartment when compared to wt controls (effect of genotype $F_{1,41}$ = 0.05; $P = 0.8227$, two-way ANOVA).

I In the tail suspension test, transgenic mice were more active than wt controls. The effect was independent of treatment (effect of genotype $F_{1,44}$ = 35.16; $P < 0.0001$, two-way ANOVA). The Bonferroni test showed a significant difference between genotypes both in vehicle and spironolactone-treated groups (***$P < 0.0001$ and **$P = 0.0067$, respectively).

J *Nrg1*-tg animals displayed an increased activity in the Y-maze test as they performed more arm choices (effect of genotype $F_{1,44}$ = 14.56; $P = 0.0004$, two-way ANOVA), with *$P = 0.0301$ in vehicle-treated and *$P = 0.0127$ in spironolactone-treated groups (Bonferroni test). There was no effect of treatment ($F_{1,44}$ = 0.39; $P = 0.5367$, two-way ANOVA).

K Naïve *Nrg1*-tg mice did not display impairments in the fear conditioning test. However, transgenic animals treated with spironolactone showed a tendency toward lower freezing rate during contextual memory test ($P = 0.0885$; Mann–Whitney test). A two-way ANOVA yielded a substantial, but not significant, interaction in the contextual memory test ($F_{1,44}$ = 3.56: $P = 0.0657$); but neither an effect of genotype ($F_{1,44}$ = 0.96; $P = 0.3315$) nor of treatment ($F_{1,44}$ = 1.16; $P = 0.2880$) was detected. Naïve *Nrg1*-tg showed an increased freezing rate during baseline recordings prior to the cue test ($^{\#\#}P = 0.0351$, Mann–Whitney test); this difference between the genotypes was not observed in groups treated with spironolactone.

L In the hot plate test, *Nrg1*-tg mice showed an increased latency to the first reaction (effect of genotype $F_{1,44}$ = 12.47: $P = 0.0010$; two-way ANOVA), which was most prominent in the group treated with spironolactone (**$P = 0.0024$, Bonferroni test) but not in vehicle-treated groups ($P = 0.2662$, Bonferroni test).

M Spironolactone treatment did not influence PPI in wt mice (effect of treatment $F_{1,22}$ = 0.00; $P = 0.9461$, two-way ANOVA).

Data information: Data are shown as mean, and error bars represent SEM; n.s., not significant; Spiro, spironolactone; pre-Spiro, data collected before spironolactone treatment. $n = 12$ per genotype and treatment with an exception in (F): *Nrg1*-tg vehicle, $n = 11$; *Nrg1*-tg Spiro, $n = 10$; wt vehicle, $n = 12$; wt Spiro, $n = 12$).

 © 2017 The Authors

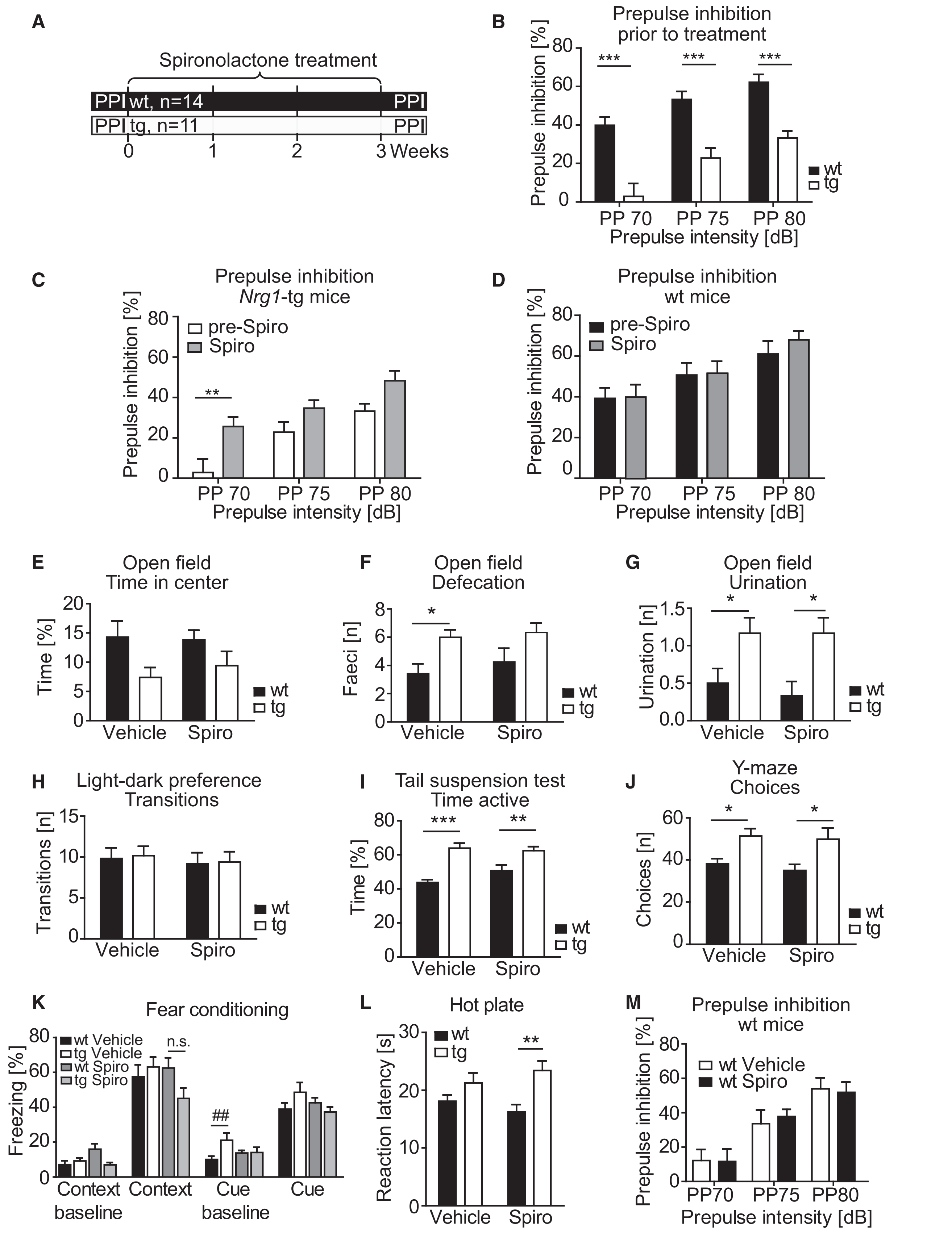
A
Spironolactone treatment
PPI wt, n=14 PPI
PPI tg, n=11 PPI
0 1 2 3 Weeks
B
Prepulse inhibition
prior to treatment
Prepulse inhibition [%]
Prepulse intensity [dB]
PP 70 PP 75 PP 80
wt
tg
C
Prepulse inhibition
Nrg1-tg mice
pre-Spiro
Spiro
D
Prepulse inhibition
wt mice
E
Open field
Time in center
Time [%]
Vehicle Spiro
F
Open field
Defecation
Faeci [n]
G
Open field
Urination
Urination [n]
H
Light-dark preference
Transitions
Transitions [n]
I
Tail suspension test
Time active
Time [%]
J
Y-maze
Choices
Choices [n]
K
Fear conditioning
wt Vehicle
tg Vehicle
wt Spiro
tg Spiro
n.s.
##
Freezing [%]
Context baseline
Context
Cue baseline
Cue
L
Hot plate
Reaction latency [s]
M
Prepulse inhibition
wt mice
wt Vehicle
wt Spiro
PP70 PP75 PP80
Prepulse intensity [dB]

Figure EV5.
