# Supplementary figures and images for: Spironolactone is an antagonist of NRG1‐ERBB4 signaling and schizophrenia‐relevant endophenotypes in mice
Source: EMBO Mol Med. 2017 Jul 25;9(10):1448–62. doi: 10.15252/emmm.201707691 (PMC5653977; doi:10.15252/emmm.201707691)

Source Data: Figure EV3A

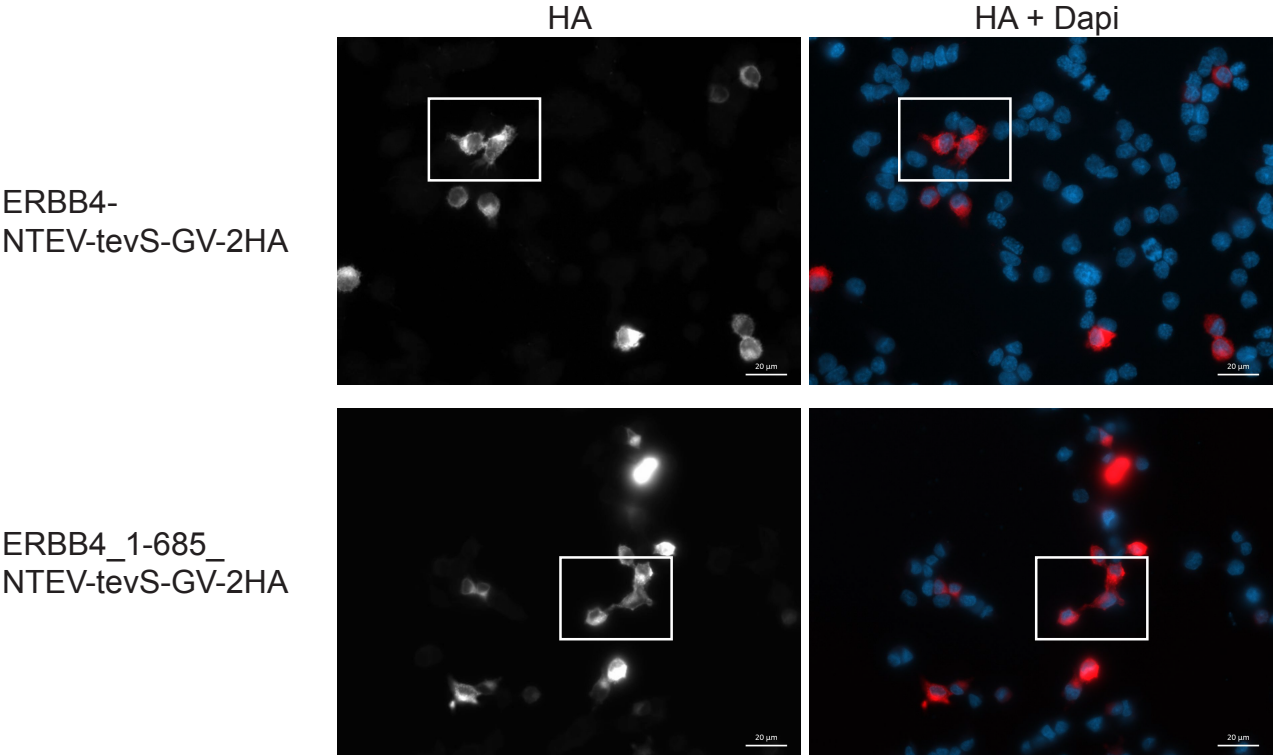

Source Data: Figure EV3D

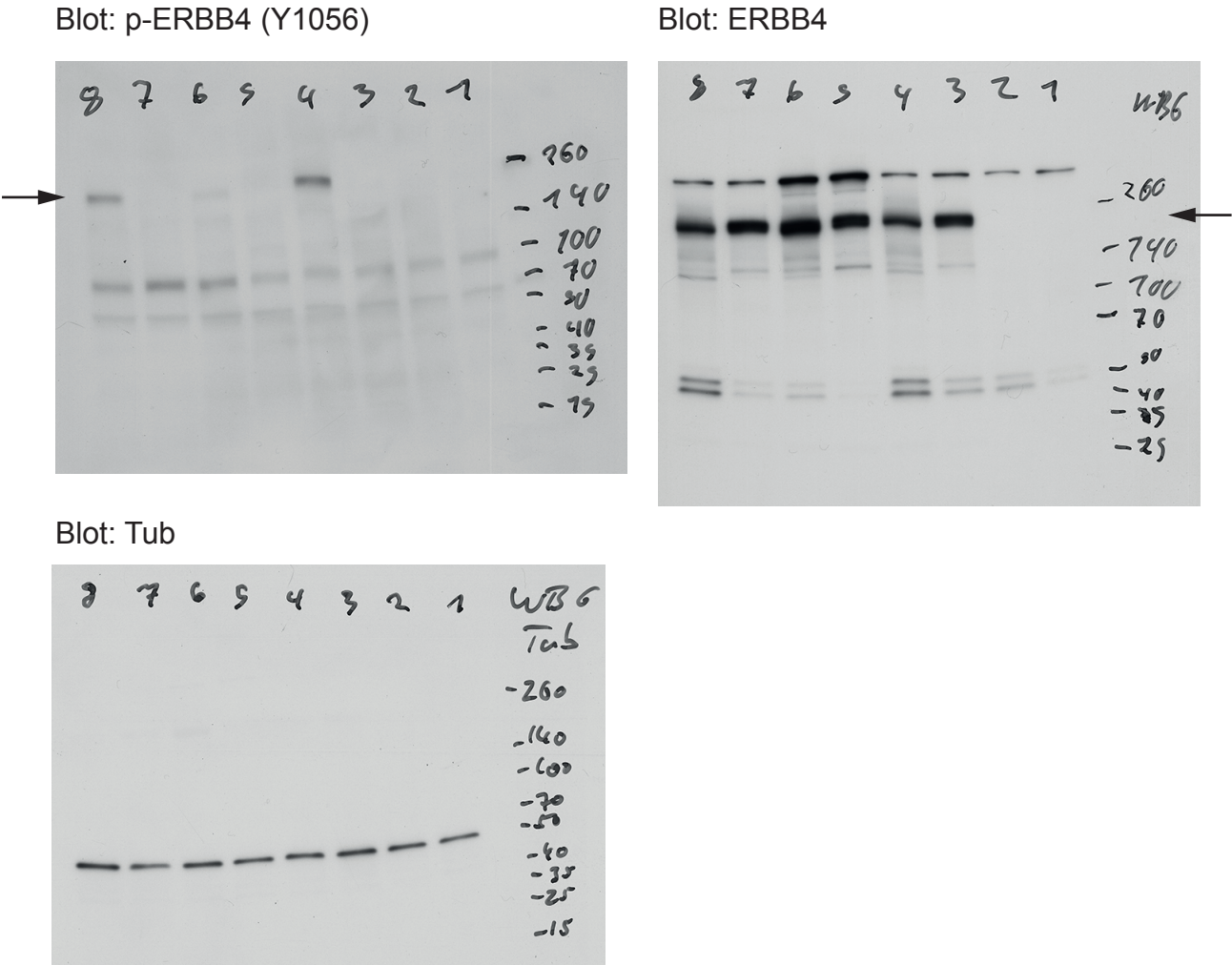

Supplement: Supplementary file 4 — Source Data for Expanded View and Appendix [file EMMM-9-1448-s005.zip › Source_Data_EV_and_Appendix/Source Data_Fig_EV3.pdf]

Source Data: Appendix Figure S2

Blot: Egfr

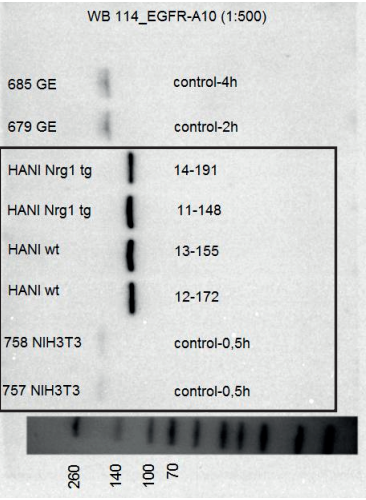

Blot: Nrg1

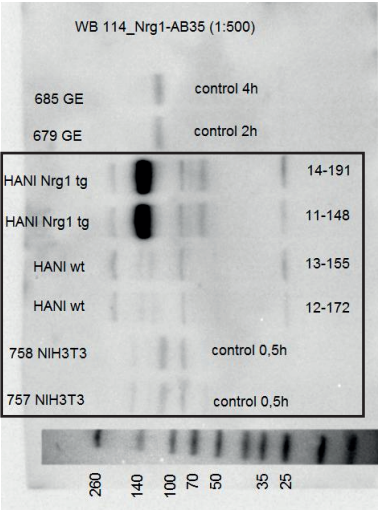

Blot: Tub

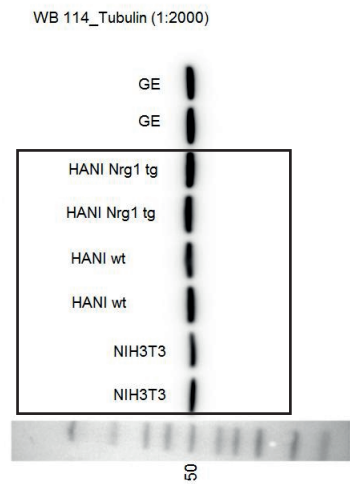

Supplement: Supplementary file 4 — Source Data for Expanded View and Appendix [file EMMM-9-1448-s005.zip › Source_Data_EV_and_Appendix/Source_Data_Appendix_Fig_S2.pdf]

Source Data: Figure EV1A

Blot: Nrg1

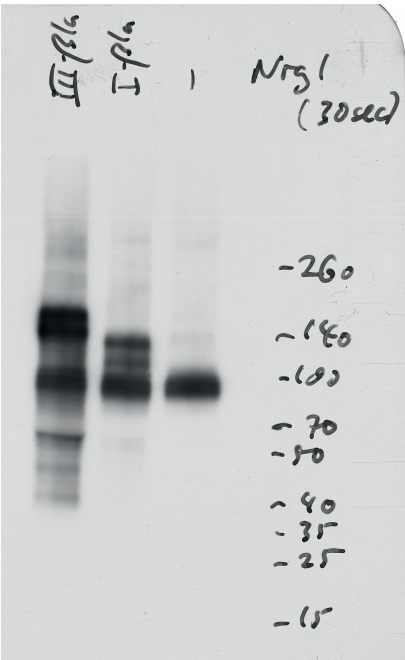

Blot: Tub

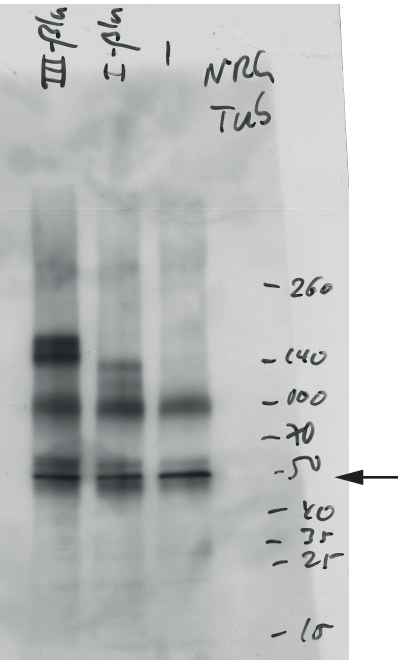

Source Data: Figure EV1B

EYFP

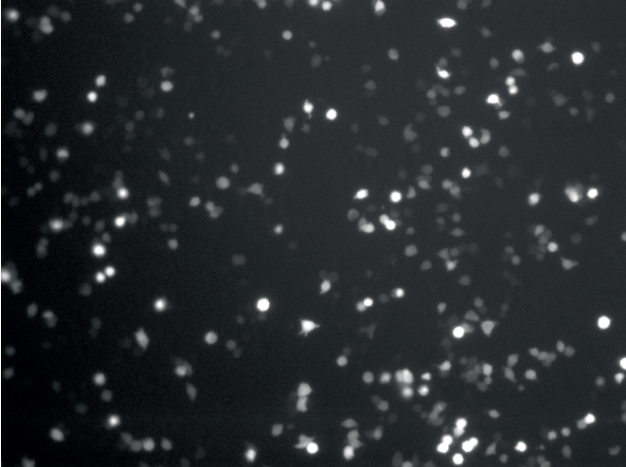

ECFP-nuc

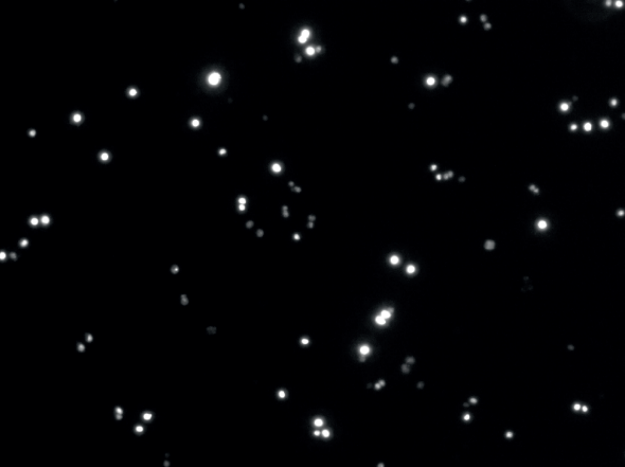

Phase contrast

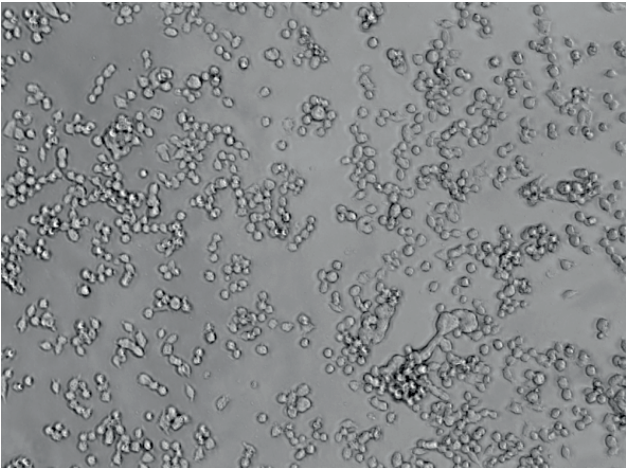

Supplement: Supplementary file 4 — Source Data for Expanded View and Appendix [file EMMM-9-1448-s005.zip › Source_Data_EV_and_Appendix/Source_Data_Fig_EV1.pdf]
