# Supplementary material for: Spironolactone is an antagonist of NRG1‐ERBB4 signaling and schizophrenia‐relevant endophenotypes in mice
Source: EMBO Mol Med. 2017 Jul 25;9(10):1448–62. doi: 10.15252/emmm.201707691 (PMC5653977; doi:10.15252/emmm.201707691)

# Source Data: Figure 4A

## Blot: p-ERBB4 (Y1056)

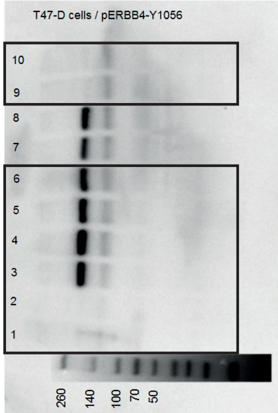


## Blot: p-ERBB4 (Y1284)

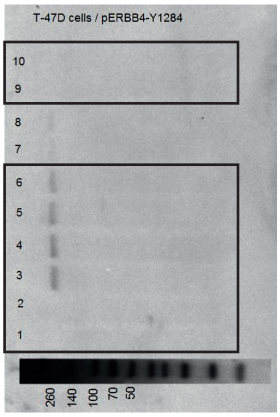


## Blot: ERBB4

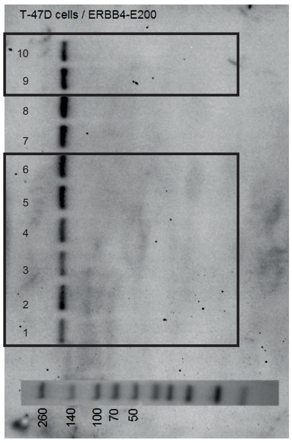


## Blot: Tub

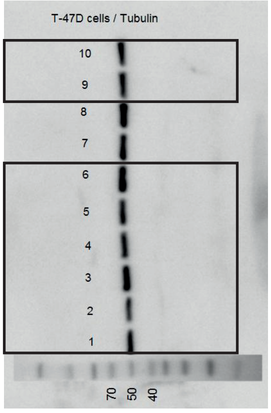


# Source Data: Figure 4D

## Blot: p-Erbb4 (Y1284)

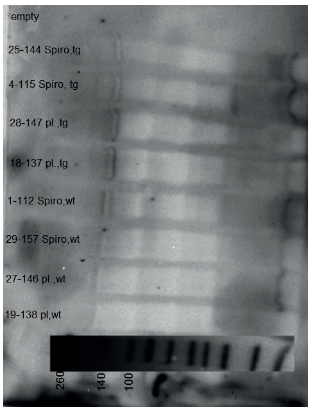


## Blot: Erbb4

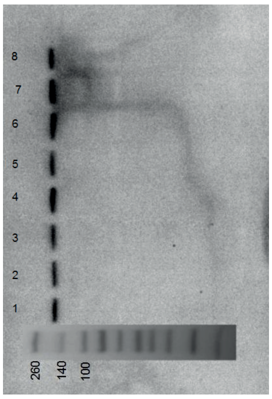


## Blot: Nrg1

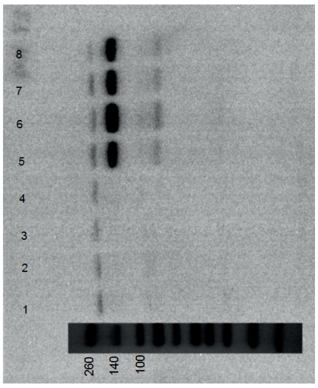


## Blot: p-Limk1 (T508)

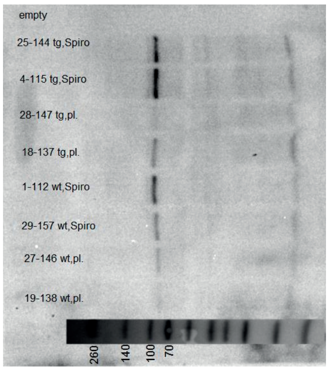


## Blot: Limk1

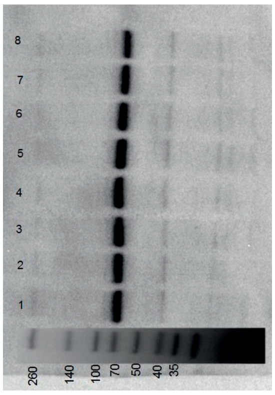


## Blot: p-Erk1/2 (T202/Y204)

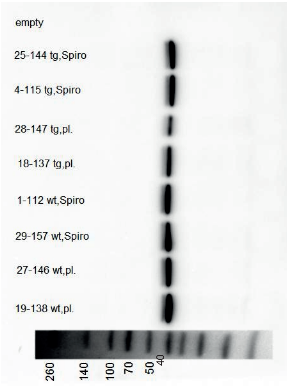


## Blot: Erk1/2

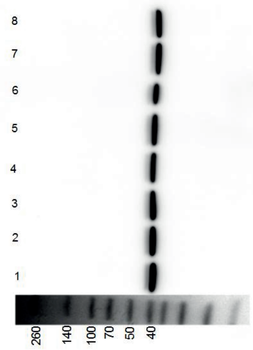


## Blot: p-Akt (S473)

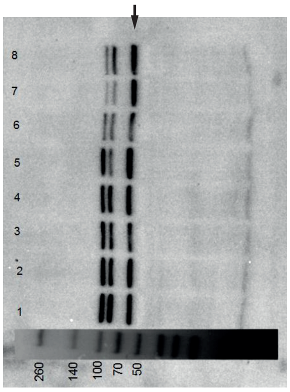


## Blot: Akt

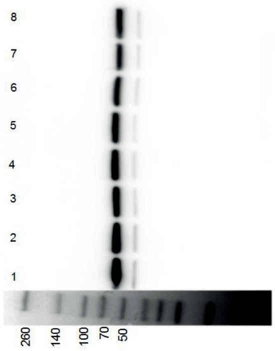


## Blot: Tub

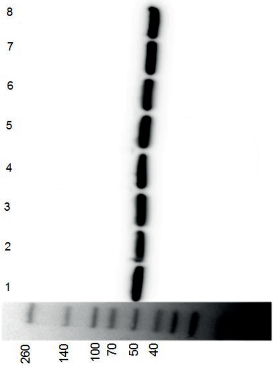

Supplement: Supplementary file 6 — Source Data for Figure 4 [file EMMM-9-1448-s004.pdf]
